# Regression Analysis to Identify Factors Associated with Urinary Iodine Concentration at the Sub-National Level in India, Ghana, and Senegal

**DOI:** 10.3390/nu10040516

**Published:** 2018-04-21

**Authors:** Jacky Knowles, Roland Kupka, Sam Dumble, Greg S. Garrett, Chandrakant S. Pandav, Kapil Yadav, Ndeye Khady Touré, Esi Foriwa Amoaful, Jonathan Gorstein

**Affiliations:** 1 Iodine Global Network, Ottawa, ON K1N 5C8, Canada; 2 United Nations Children’s Fund, New York, NY 10017, USA; rkupka@unicef.org; 3 Statistics for Sustainable Development, Reading RG1 4QS, UK; s.dumble@stats4sd.org; 4 Global Alliance for Improved Nutrition, 1202 Geneva, Switzerland; ggarrett@gainhealth.org; 5 All India Institute of Medical Sciences, New Delhi 110029, India; cpandav@gmail.com (C.S.P.); dr.kapilyadav@gmail.com (K.Y.); 6 Cellule de Lutte contre la Malnutrition, B.P. 45001 Dakar-Fann, Senegal; ndeyekhadytoure@yahoo.fr; 7 Nutrition Department, Ghana Health Service, Accra, Ghana; esiforiwa@gmail.com; 8 Iodine Global Network, Seattle, WA 98107, USA; jgorstein@ign.org

**Keywords:** iodine deficiency, iodised salt, single-variable regression, multiple-variable regression, urinary iodine

## Abstract

Single and multiple variable regression analyses were conducted using data from stratified, cluster sample design, iodine surveys in India, Ghana, and Senegal to identify factors associated with urinary iodine concentration (UIC) among women of reproductive age (WRA) at the national and sub-national level. Subjects were survey household respondents, typically WRA. For all three countries, UIC was significantly different (*p* < 0.05) by household salt iodine category. Other significant differences were by strata and by household vulnerability to poverty in India and Ghana. In multiple variable regression analysis, UIC was significantly associated with strata and household salt iodine category in India and Ghana (*p* < 0.001). Estimated UIC was 1.6 (95% confidence intervals (CI) 1.3, 2.0) times higher (India) and 1.4 (95% CI 1.2, 1.6) times higher (Ghana) among WRA from households using adequately iodised salt than among WRA from households using non-iodised salt. Other significant associations with UIC were found in India, with having heard of iodine deficiency (1.2 times higher; CI 1.1, 1.3; *p* < 0.001) and having improved dietary diversity (1.1 times higher, CI 1.0, 1.2; *p* = 0.015); and in Ghana, with the level of tomato paste consumption the previous week (*p* = 0.029) (UIC for highest consumption level was 1.2 times lowest level; CI 1.1, 1.4). No significant associations were found in Senegal. Sub-national data on iodine status are required to assess equity of access to optimal iodine intake and to develop strategic responses as needed.

## 1. Introduction

Iodine deficiency is the world’s most prevalent cause of preventable brain damage. The most severe, visible, forms of the deficiency are now rare due to prevention through Universal Salt Iodisation (USI), which is accepted as a highly cost-effective public health strategy [1]. However, where salt iodisation is not universal, mild to moderate deficiency still occurs, often resulting in invisible impairment to foetal brain development, child mental capacity and cognitive function, and the related impact on population development [2].

Since the 1990 World Health Assembly resolution on the elimination of iodine deficiency [3], many countries have implemented mandatory legislation for salt iodisation and as a result, 86% of the world’s households now consume iodised salt [4,5]. Consistent with the basis of a 1994 UNICEF (United Nations Children’s Fund)-WHO joint committee statement [1], this increased access to iodised salt has been accompanied by a decrease in the number of countries with iodine deficiency (defined as a national median urinary iodine concentration (MUIC) of below 100 µg/L among school-age children or, where data for children are unavailable, among women of reproductive age) from more than 110 to 20 countries between 1993 and 2017 [6].

Despite the success in terms of national status, sub-national areas of iodine deficiency remain within some countries. These areas have generally, but not exclusively, been associated with indicators of lower socioeconomic status and low coverage of adequately iodised salt [7,8]. Currently there is no strong evidence base for where iodine deficiency still exists at the sub-national level, or for which factors are most associated with (sub)-population urinary iodine concentration in different contexts. Alongside this, there is growing recognition of the dietary shift away from the use of household/cooking salt to intake of salt-containing foods and condiments [9,10,11], such as bouillon, which could weaken the association between iodine status and the iodine content of household salt. These evidence gaps present a challenge to the design of strategies to ensure that optimal dietary iodine intake is achieved among all sub-national population groups. Therefore, we assessed the key factors associated with population iodine status among women of reproductive age at the national and sub-national level, using data from iodine surveys conducted in India, Ghana, and Senegal. The surveys were conducted in parallel using similar tools and were designed to inform sub-national USI strategies, while also providing updated data on iodine status. All three countries have mandated the iodisation of household salt, introduced during the period from 1994 (Senegal) to 2005 (Ghana) [12,13,14].

## 2. Materials and Methods

### 2.1. Survey Design

The surveys were cross-sectional and adopted a stratified, multistage cluster sample design. They were conducted during the period from December 2014 to April 2015. Surveys were designed to provide representative information by sub-national domains (strata), determined according to their administrative or programmatic relevance. Probability proportional to size (PPS) methodology was used to select primary sampling units (PSUs) with systematic random selection of the required number of households within each PSU. The questionnaire was designed to include indicators thought to be associated with access to adequately iodised household salt along with information about consumption of certain processed foods and condiments that typically contribute to dietary salt, and potentially iodine intake.

The primary aim of each survey was to assess household iodised salt coverage, therefore, the target unit for all surveys was the household. The first choice of respondent was a woman responsible for organizing food preparation in the household, usually the wife of the head of household, or the head of the household. The second-choice respondent was a woman of reproductive age (WRA). Although a WRA was defined as being 15–49 years of age, women aged 18 or above were preferentially selected to respond to complete the household interview. Where nobody meeting these criteria was present in the household, then a man was selected as the respondent.

Stratification in Ghana and Senegal aimed to obtain data representative of small-scale salt production areas that were expected to have low household coverage of adequately iodised salt and potentially lower urinary iodine concentration (UIC). The India survey was the first national survey to include quantitative assessment using the iodometric titration method of household iodised salt use and assessment of sodium intake and population iodine status. Stratification in India was, therefore, by urban and rural areas within six defined geographical zones: South, West, Central, North, East, and North-East (12 strata total).

### 2.2. Survey Administration and Field Procedures

Interviews were conducted in all selected and consenting households. Interviews were conducted under the supervision of experienced field supervisors, with technical personnel at a central administrative level available to assist with coordination and troubleshooting. In-depth training was conducted prior to the surveys, and survey tools and procedures were pilot-tested in a typical field setting. There was no replacement of households where a respondent was not at home or the potential respondent refused to participate.

A random spot urine sample was collected from consenting non-pregnant WRA who were selected according to the different survey protocols. These protocols were agreed upon based on a balance between the expected number of WRA per household, survey resources, and the desired level of precision around the stratum-level MUIC value. All results presented for WRA in this paper refer to non-pregnant WRA.

Urine samples were collected in a sterile container, according to instructions provided by the interviewer. Containers were barcode-labelled with a unique ID that linked with the ID number for the household and the salt sample. In some cases, urine was transferred to smaller (sterile, bar-coded) tubes before transporting to the laboratory. In all cases, samples were placed in one container for each PSU, which was barcode-labelled with the unique PSU number, and transported on ice to the designated laboratory for analysis of iodine.

A sample of approximately 50 g of salt was targeted for collection from all consenting households in each survey. Salt samples for each household were stored in re-sealable bags barcoded with the unique household identification number. Bags for all household salt samples from one PSU were stored in a barcode-labelled opaque bag/envelope at room temperature until analysis of iodine content at the laboratory.

Questionnaire data were collected using mobile data collection forms with pre-coded skips and cross-checks to ensure data quality. Completed forms were checked in the field then uploaded daily, or at the earliest possible opportunity. Summary reports on the uploaded data were compiled and shared with the survey principal investigator three times a week, highlighting any unexpected findings/missing data and/or lower than expected response rates for any component.

### 2.3. Indicators/Survey Tools

All survey instruments captured classification of residence type (urban vs. rural); respondent consent; and collection of urine and household salt samples. Furthermore, all survey tools included modules to determine the household Multidimensional Poverty Index (MPI) score [15,16]; respondent’s awareness of iodine deficiency and of iodised salt (having ever heard of either); the household’s typical salt purchasing behaviour, dietary diversity of the respondent, and (in Ghana and Senegal) frequency of consumption of specific foods considered to contribute to salt intake across different population groups.

The MPI was comprised of three domains: health, education, and living standards; each domain was comprised of sub-components. A higher score for each domain was equated with greater vulnerability to poverty for that component. Frequency of consumption of specific foods was based on questions to a respondent WRA about the estimated number of days in the past week they had consumed the food. Where the food had been consumed in the past week, a following question was asked about the average number of times a day the product was consumed. In Ghana the salt-containing foods included for frequency of consumption questions were bouillon, tomato paste, and instant noodles, and in Senegal it was bouillon.

The methodology used to assess a woman’s individual dietary diversity score was adapted from the Food and Nutrition Technical Assistance project (FANTA) and the Food and Agriculture Organization of the United Nations (FAO) models [17,18], which assess the number of different food groups consumed in the past 24 h. Responses for both food frequency and dietary diversity questions were not necessarily provided by the same WRA who provided the urine sample for iodine analysis, however, for these analyses, the respondent’s answers were used as a proxy for other WRA in the household.

Height and weight measurements were recorded for WRA in the India survey only, allowing for calculation of body mass index (BMI).

The primary outcome variable for each survey was the national and stratified coverage of adequately iodised salt, defined as the percent of households using salt with 15 or more mg iodine/kg of salt. Results for household iodised salt use are reported elsewhere [19]. The secondary outcome indicator, included in the models for this paper, was the national and stratified median urinary iodine concentration (MUIC) among WRA. Questions to determine whether the WRA providing a urine sample was pregnant or not were included in each survey and data from pregnant or possibly pregnant women were excluded from these analyses.

According to global guidance, a MUIC above 100 µg/L indicates adequate iodine intake among populations of WRA, while a median above 199 µg/L indicates iodine intake above requirements [20].

Salt was categorised as non-iodised, where the iodine content was <5 mg/kg (to account for naturally occurring trace levels of iodine); inadequately iodised, where the content was between 5 and 14.9 mg/kg; and adequately iodised, where the iodine content was 15 mg/kg or above [20].

### 2.4. Determination of Urinary and Salt Iodine Content

Urinary iodine concentration was determined using ammonium persulfate digestion with spectrophotometric detection of the Sandell-Kolthoff reaction in Ghana and Senegal [21], and using the modified microplate process for this method in India [22]. Both methods are comparable and both have proved to have acceptable performance and accuracy [23]. Salt iodine was assessed using the iodometric titration method [20]. An external quality-assurance network was established for the duration of these three surveys, with a third-party laboratory (Uttar Pradesh State USI Coalition Technical Laboratory, Sanjay Gandhi Postgraduate Institute of Medical Sciences, Lucknow, India) providing periodic internal and external urine and salt samples to verify the methodology and satisfactory performance of the laboratories. In addition, all three laboratories were participating to different extents in the Ensuring the Quality of Urinary Iodine Procedures (EQUIP) program of the US Centers for Disease Control and Prevention [23] at the time of the survey.

### 2.5. Data Management

Initial survey data management and analysis was conducted by the Statistical Services Centre, University of Reading, United Kingdom. All data were adjusted for the relative proportion of the population in each stratum.

Each MPI domain was given an equal weight (one-third) in determining the overall MPI score. A household was classified as being deprived in any one domain if the score for that domain was greater than or equal to 0.3 (scale of 0 to 1). Where the overall MPI score (based on adjustments to give one third weighting to the scores for each domain) was greater than or equal to 0.3, the household was considered as being vulnerable to poverty.

Weekly consumption of bouillon, tomato paste, and instant noodles were estimated based on the reported number of days per week consumed, multiplied by the average number of times per day they were consumed. Typical serving sizes were used to convert these frequencies into gram intake for bouillon (serving size of 1.25 g, personal communication Abdul-Razak Abizari, University for Development Studies, Tamale, Ghana), tomato paste (serving size of 30 g, based on product label information), and instant noodles (serving size of 85 g based on average packet weight). The related salt intake from these foods was then estimated based on typical salt content reported on the food label or through food industry sources: 0.5 g salt/1 g bouillon, 0.1 g salt/1 g tomato paste, and 3 g salt per 85 g packet instant noodles [24]. A similar approach was taken to estimate consumption and salt content in previous studies [11].

For the regression analyses, the outcome variable, UIC values, were log transformed for calculation of means. Intake of bouillon and tomato paste during the previous week was assigned to one of three categories. For bouillon: 0 g, 1–19 g, and ≥20 g; for tomato paste: 0 g, 1–199 g, and ≥200 g. Instant noodle intake was categorised as yes or no.

The individual dietary diversity scores ranged from 0 to 9 based on 9 different food group categories. A score of <4 was categorised as a non-diverse diet [17].

### 2.6. Data Analysis

The variables included in the models for each country are those listed in the first column of respective, country data, tables. All variables were treated as categorical, with the categories matching those given in the results tables. Reference levels are also indicated in the tables as the first category mentioned in each of the categorical variables.

The regression analyses were conducted using different models, one that included the overall MPI score and one using the three domain-specific MPI scores for education, health, and living standards. The final model included the domain-specific MPI scores and not the overall MPI score because this allowed for more refined analysis of factors associated with urinary iodine concentration and any interaction with strata. All analyses were conducted separately for each country, using standardised variable and category definitions where possible. Results for urinary iodine concentration are presented as the median with interquartile range (IQR) and mean with 95% confidence intervals (CI) for all non-pregnant WRA who consented to provide a urine sample and had valid urinary iodine results. All results presented are adjusted for survey design effects.

Urinary iodine concentration was analysed against multiple different factors using general linear models, with household weights and robust variance estimation accounting for survey design effects using the survey library “survey: analysis of complex survey samples” within the R statistical analysis package version 3.31 [25]. *p*-values presented are from the Wald test determining the overall significance of each variable.

#### 2.6.1. Single Variable Regression Analysis

*p*-values (based on the mean of the log transformed UIC) are not adjusted for multiple comparisons; many of the factors considered are explicitly non-independent (e.g., Strata and urban/rural) so a naïve adjustment measure would not be appropriate. However, the number of tests being conducted should be taken into consideration when considering the significance of variables with borderline *p*-values, between 0.01 and 0.05.

#### 2.6.2. Multiple Variable Regression Analysis

All variables included in the single variable analysis were considered for inclusion in the models except for residence type where this was included in the definition of the strata, as was the case in India and Senegal. A stepwise selection procedure was conducted using a *p*-value of less than 0.1 as the inclusion criteria. An additional model was run, including interaction by strata to investigate if and how associations between household salt iodine content and urinary iodine level varied by strata in Ghana and Senegal. For India, the number of WRA from households using non-iodised salt was too low to generate reliable estimates of average urinary iodine concentration for each of the 12 strata, therefore the analysis was conducted including interactions by urban and rural residence type.

Further details of individual survey design, tools, and data management, adjustments, and analysis can be found in the full survey reports [26,27] (personal communication from Ghana Health Services on the unpublished draft survey report) and an associated publication [28].

The national surveys were approved by national or academic Institutional Review Boards in each of the three countries. All protocols required consent for the interview and for collection of a salt and urine sample.

## 3. Results

An overview of the survey design and outcome, including the protocol for selection of WRA, response rates for interview and for urinary iodine analysis, and the respondent characteristics (sex and age) are shown for each of the three countries in Table 1.

The number of target households for interview and salt collection per strata varied from 504 in India, with 252 households targeted for urine collection from all consenting WRA in those households; to 528 in Ghana, with all households targeted for urine collection from all consenting WRA; and 656 in Senegal, with all households targeted for urine collection from one consenting WRA. Response rates were over 90% for completed interviews in all countries. Response rates for urinary iodine analyses were 86.5% in India, 79.9% in Ghana, and 85.4% in Senegal. Numbers of urine samples excluded from the analysis due to reporting being pregnant or possibly pregnant are given in the footnote to Table 1. The percent of urine samples provided by a WRA who was also the respondent for the dietary diversity and food frequency questions was 80.2% in India, 74.2% in Ghana, and 85.5% in Senegal. The mean age of WRA providing urine samples was 31.7 years in India, 30.5 years in Ghana, and 28.3 years in Senegal.

Unless otherwise indicated, data for all results discussed below are shown in Table 2, Table 3 and Table 4 for India, Ghana, and Senegal, respectively.

### 3.1. Iodine Status

Iodine status among the population of WRA in each stratum is shown as the MUIC in Table 2, Table 3 and Table 4. Iodine status was adequate among WRA from all strata in the India survey. The lowest (but still adequate) MUIC of 114.7 µg/L was reported for WRA in the Central-rural strata. In Ghana, the MUIC by strata ranged from 168.0 µg/L among WRA in the North to 316.3 µg/L among WRA in the South non-salt-producing areas, indicating iodine intake above requirements in this stratum. In Senegal the MUIC reflected much poorer iodine status among WRA across the population. The lowest median of 82.9 µg/L was found among WRA in the rural non-salt-producing-areas. The highest median of 111.9 µg/L was found among WRA in the urban stratum.

### 3.2. Single Variable Regression Analyses

Single variable regression analysis showed that urinary iodine concentration was significantly different between strata in India and Ghana (*p* < 0.001 for both), but not in Senegal. In India and Ghana, WRA from urban areas tended to have better iodine status than WRA from rural areas (*p* = 0.002 and *p* = 0.008 respectively), however the MUIC indicated adequate status among WRA with both urban and rural residency in both countries. There was a trend towards the same difference by residence type in Senegal, however it did not reach significance (*p* = 0.074). The MUIC of 82.9 µg/L for WRA in the two combined rural areas indicated that this population was iodine deficient.

A significant positive association between UIC among WRA and household salt iodine category was found in all three countries (*p* < 0.001 in India and Ghana, *p* = 0.043 in Senegal). In Senegal this was the only variable significantly associated with UIC, and an adequate iodine status (MUIC of 123.1 µg/L) was only achieved among the population of WRA in households with adequately iodised salt (≥15 mg/kg iodine). WRA from households using non-iodised and inadequately iodised salt had significantly lower MUICs of 83.8 µg/L and 84.9 µg/L respectively.

With regard to differences in UIC by MPI component domains, significantly higher UIC was found among WRA in India from households that were non-deprived for the MPI Living Standards domain (*p* < 0.001). See Table 2. In Ghana, WRA from households categorised as non-deprived in each of the three MPI domains had higher UIC than WRA from deprived households for the respective domain (*p* = 0.002 for MPI Education and MPI Living Standards, *p* = 0.008 for MPI Health). See Table 3.

WRA who had heard of iodine deficiency tended to have higher UIC than WRA who had not heard of iodine deficiency, however this association was only significant in India (*p* < 0.001). Other significant single variable analysis associations with UIC found in India were by dietary diversity and BMI (*p* = 0.001 and *p* = 0.036 respectively). WRA with diverse diets had a MUIC of 175.1 µg/L and WRA with a non-diverse diet had a MUIC of 150.8 µg/L. While WRA with low BMI (<18.5 kg/m^2^) had a MUIC of 145.3 µg/L, compared to MUICs of 161.4 µg/L and 161.7 µg/L for WRA with normal and high BMI, respectively.

Other significant differences in UIC were found in Ghana, by frequency of consumption of tomato paste (*p* < 0.001) and bouillon (*p* = 0.003). WRA who had not consumed any tomato paste in the previous week had significantly lower UIC, with a MUIC of 163.0 µg/L, than WRA who had consumed 1–200 g or >200 g (respective MUICs were 210.6 µg/L and 196.8 µg/L). The association between UIC and reported bouillon intake, although significant overall, did not show a specific trend: WRA reporting to have the highest intake of bouillon in the previous week (>20 g) had a lower UIC, MUIC of 167.5 µg/L, than WRA reporting to have not consumed bouillon, MUIC of 200.3 µg/L. The highest UIC, with corresponding MUIC of 216.2 µg/L, was found among WRA who had an estimated consumption of between 1 g and 20 g bouillon in the previous week.

### 3.3. Multiple Variable Regression Analyses

In multiple variable regression analyses UIC was strongly associated with strata and with household salt iodine content in India and Ghana (*p* < 0.001) (Table 2 and Table 3). No variables were found to be significantly associated with UIC among WRA from the multiple variable regression model in Senegal (Table 4).

In India, the only individual stratum where UIC among WRA was significantly higher than among WRA in the reference region of South-urban was the North–urban area (*p* < 0.001). Using the back-transformed coefficients from the regression models the estimated iodine was 1.5 times greater in the South-urban area than the North-urban area (95% CI 1.2, 1.9).

In Ghana, estimated UIC tended to be higher among WRA in the two Southern regions when compared with status among WRA in the North and mid regions, however, there were no significant pairwise differences relative to the reference stratum of the South-salt-producing region.

In multiple variable models, estimated UIC increased significantly with increasing household salt iodine content in India and Ghana. The reference group for these comparisons was WRA from households using non-iodised salt. The estimated UIC among WRA from households using inadequately- and adequately-iodised salt in India was 1.3 (95% CI 1.1, 1.6) and 1.6 (95% CI 1.3, 2.0) times higher relative to the reference. In Ghana, the estimated UIC among WRA in households using inadequately iodised salt was not significantly different to the reference group, however the estimated UIC among WRA from households using adequately iodised salt was 1.4 (95% CI 1.2, 1.6) times higher than that in the reference group.

In India, other significant positive associations for estimated UIC among WRA after holding other covariates constant, were found for WRA with diverse diets, UIC 1.1 (95% CI 1.0, 1.2) times higher than among WRA with non-diverse diets; and for WRA from households where the respondent had heard of iodine deficiency, UIC 1.2 (95% CI 1.1, 1.3) times higher than among WRA from households where the respondent had not heard of iodine deficiency.

In Ghana, the other significant positive association with estimated UIC found from multiple regression analysis, was with increasing consumption of tomato paste (*p* = 0.029). WRA who had an estimated consumption of at least 200 g tomato paste in the previous week had an estimated UIC 1.2 (95% CI 1.1, 1.4) times higher than among WRA who had not consumed any tomato paste in the previous week. The association between adjusted estimated UIC and consumption of bouillon was also significant (*p* = 0.023), however, as found for the single variable analysis, the positive association with iodine status was only seen for WRA consuming no bouillon (reference group) compared with some but less than 20 g bouillon in the previous week, where estimated UIC was 1.2 (95% CI 1.1, 1.3) times higher. Estimated UIC among WRA consuming over 20 g bouillon in the previous week was not significantly different to that in the reference group.

**Figure 1:**
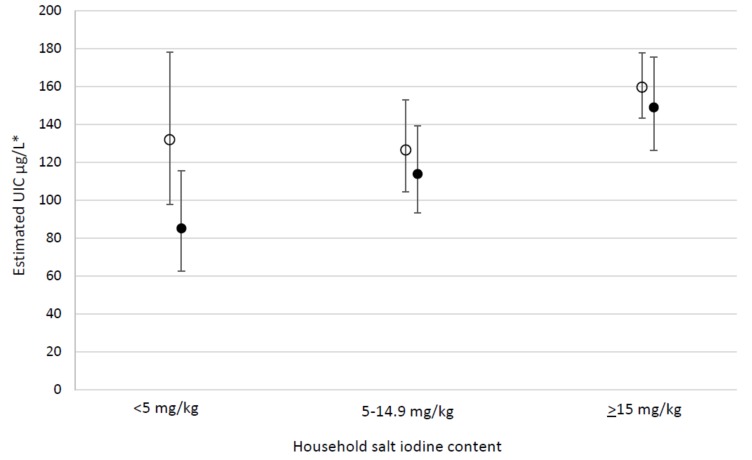
Multiple regression analysis including interaction with urban/rural residence type, to estimate predicted UIC (µg/L) among WRA according to household salt iodine category (mg/kg) in India. ○ Urban, ● Rural. * Adjusted estimates with 95% confidence intervals for urinary iodine concentration (UIC), back-transformed from a log-linear model adjusting for strata, multi-dimensional poverty index (MPI) components, awareness of iodine deficiency, dietary diversity, age, and BMI.

**Figure 2:**
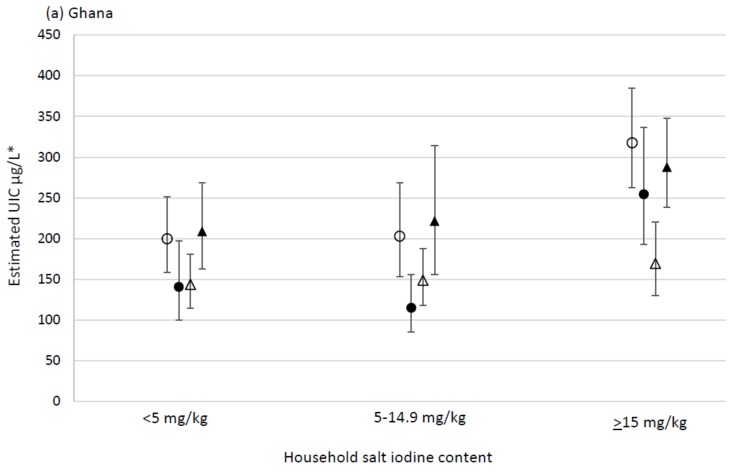

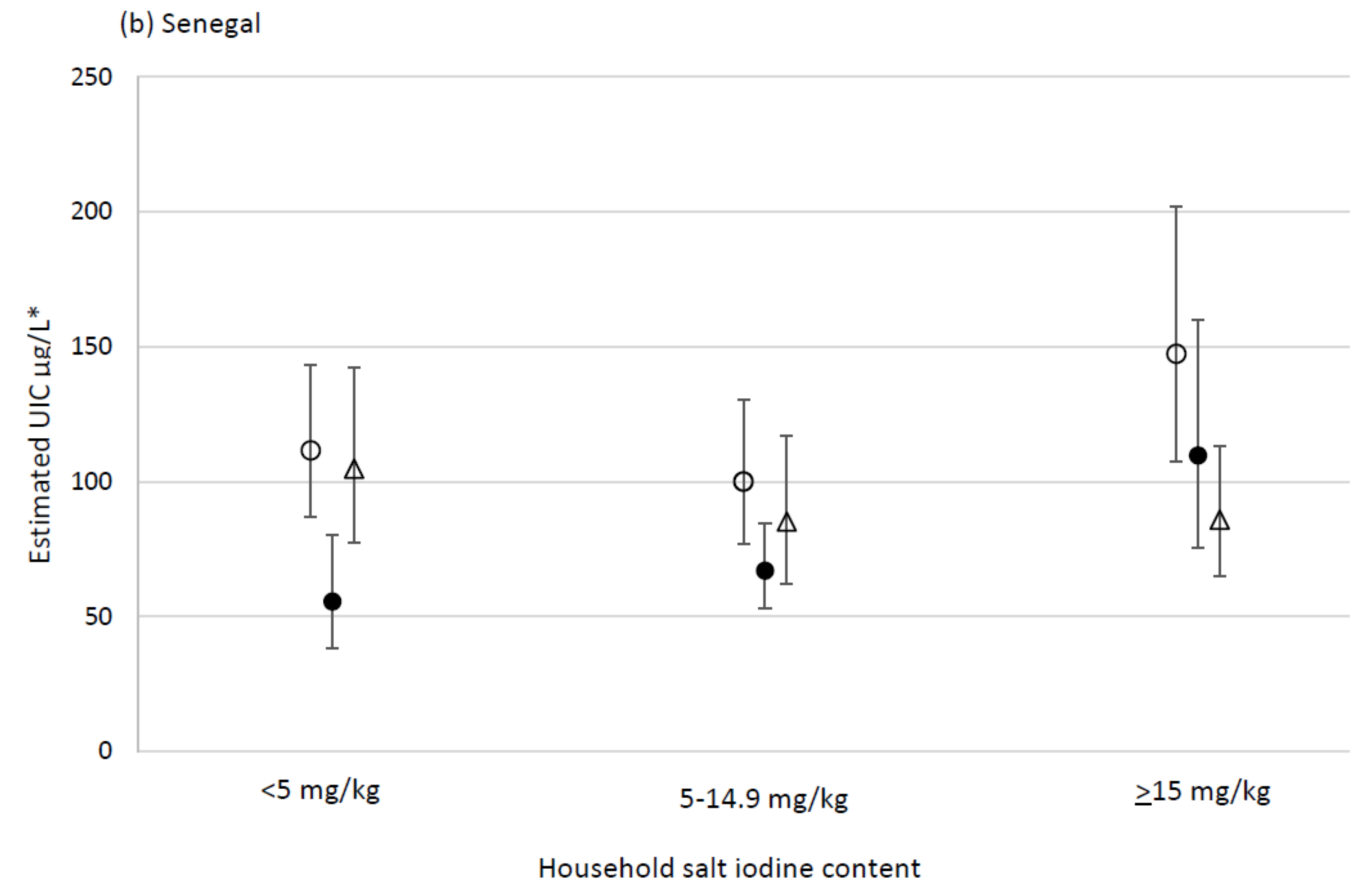
Multiple regression analysis including interaction with strata, to estimate predicted UIC (µg/L) among WRA according to household salt iodine category (mg/kg) in (**a**) Ghana ○ South-salt-producing, ● North, ∆ Mid, ▲ South-non-salt-producing and (**b**) Senegal ○ Rural-salt-producing, ● Rural-non-salt-producing, ∆ Urban. * Adjusted estimates with 95% confidence intervals for urinary iodine concentration (UIC); back-transformed from a log-linear model adjusting for residence type; multi-dimensional poverty index (MPI) components; awareness of iodine deficiency; dietary diversity; age; estimated consumption of tomato paste, bouillon and instant noodles.

There was no significant interaction of the effect of residence type with strata in India (*p* = 0.145), illustrated in Figure 1. The effect of residence type varied significantly by strata in Ghana (*p* = 0.031) and Senegal (*p* = 0.023) as illustrated in Figure 2. 

In the South-salt-producing and the North strata in Ghana, the estimated UIC among WRA from households using adequately iodised salt was significantly higher (non-overlapping 95% CI) than among WRA from households using non-iodised salt in the same strata, when holding all other covariates constant. The same trend of increasing estimated UIC with increasing household salt iodine content was observed among WRA from the South-non-salt-producing stratum. However, estimated UIC among WRA in the mid stratum remained relatively similar regardless of household salt iodine category. Moreover, when holding other covariates constant, estimated UIC among WRA from households using adequately iodised salt was significantly lower for WRA in the mid stratum than for WRA in the two Southern strata. See Figure 2a.

In the two rural strata in Senegal, there was a (non-significant) trend for estimated UIC to be higher among WRA from households using adequately iodised salt than among WRA from households using inadequately or non-iodised salt, when other covariates were held constant. In the urban stratum, the highest estimated UIC was among women from households using non-iodised salt, however the number of WRA for this estimate was small (*n* = 77) and the 95% CI for the UIC estimate overlapped with estimates for WRA from households using adequately and inadequately iodised salt). See Figure 2b. Notable from the figure is that the estimate for UIC among WRA from households using non-iodised salt was significantly lower for WRA in the rural-non-salt-producing stratum than for WRA in the other two strata.

## 4. Discussion

The single variable regression analyses in this paper showed that relative UIC levels were significantly different by household salt iodine category in all three countries. Other common significant differences in UIC were found by strata and by household vulnerability to poverty in India and Ghana. In multiple variable regression analysis, UIC was significantly associated with strata and household salt iodine category in India and Ghana, with estimated UIC 1.4 to 1.6 times higher among WRA from households using adequately iodised salt than among WRA from household using non-iodised salt. Other significant associations with UIC from multiple variable regression were found in India, with having heard of iodine deficiency and having improved dietary diversity; and in Ghana, with the level of consumption of tomato paste in the previous week. No significant associations were found in Senegal.

National iodine survey results showed remarkable progress towards the achievement of optimal iodine status in India and Ghana, where adequate iodine status (MUIC > 100 µg/L) was found among all sub-populations of WRA. Meanwhile, in Senegal the national MUIC of 98.0 µg/L was below the cut off of 100 µg/L used by the WHO to indicate adequacy of iodine intake among WRA [20]. However, there is some evidence to suggest that a cut off below a MUIC of 100 µg/L among this population group would be more appropriate to define iodine adequacy [29]. Iodine status was lowest among WRA in the rural-non-salt-producing stratum where the MUIC was 82.9 µg/L, suggesting that women in this stratum may be entering pregnancy with sub-optimal iodine status, potentially affecting foetal development. The outcome of ongoing research to determine the most appropriate cut off for deficiency among WRA will help determine the level of concern around these lower population MUIC values in Senegal.

Significant differences in UIC found from single variable analyses were mostly in the direction expected based on existing evidence of factors generally associated with greater access to iodised household or processed food salt [7,30], and/or with increased exposure to native dietary iodine through increased dietary diversity, and to environmental iodine. Higher socioeconomic status households and urban locations (associations with UIC found in India and Ghana) are factors found to be associated with improved access to iodised household salt [19,31,32], increased diversity of diet and consumption of processed foods [9,10,33,34], and with environmental iodine from human activities [35]. In Ghana, the higher UIC found among WRA living in the two southern strata locations was expected given the substantially higher groundwater iodine levels found in these regions of the country [36].

Associations between estimated UIC and household deprivation (MPI) were not significant in either country after applying the multiple variable regression model, supporting the likelihood that the association between UIC and MPI components was due to the association of MPI with multiple co-related factors, including better access to iodised salt and improved dietary diversity.

In Ghana, UIC was positively associated with household salt iodine content even after controlling for estimated intake of frequently consumed foods that contribute to (potentially iodised) salt intake. Bouillon, tomato paste, and instant noodles illustrate the important role of iodine from household salt in maintaining iodine status. It may also indicate that the foods products assessed are not consistently produced with iodised salt.

India was the only country where the positive association between having heard of iodine deficiency and estimated UIC among WRA was significant, both by single and multiple variable regression analysis. This may reflect the strength of activities to raise awareness about iodised salt and change related purchasing behavior [37]. However, there is no direct connection between awareness and iodine status. It is, therefore, proposed that having heard of iodine deficiency may be associated with more than one independent situation or behaviour leading to improved iodine intake. Potential associations could be with a combination of, or all of, lower levels of deprivation, increased dietary diversity, urban residence, and increased access to adequately iodised salt.

The association between low BMI and lower UIC among WRA observed from the single variable regression model in India may be explained by an increased intake of salt, which was observed to be related to BMI in one Indian state in a separate study [38]. There is evidence that increased salt intake may be associated with increased energy intake in some groups, but could also be an independent risk factor for obesity, unrelated to energy intake [39].

In Ghana, the strong positive association between UIC and intake of tomato paste, which remained after multiple variable regression analysis, indicates that iodised salt may have been used in the production of most or all brands of tomato paste. The inconsistent association between estimated UIC and bouillon intake is more complicated and difficult to explain. The result does not suggest that iodised salt is used in the majority of bouillon products consumed, which would contrast with the findings from a study in Northern Ghana [40]. However, it may also partially reflect the difficulty in obtaining accurate reports of bouillon intake from food frequency questions, since the amount of bouillon used can vary greatly and its inclusion in certain foods might not be recognised by the consumer.

In Senegal, none of the survey variables was found to be significantly associated with UIC after multiple variable regression analysis. This lack of association possibly reflected the overall low iodine status, indicating few sources of iodised salt or other iodine in the diet, and a relatively low variation in UIC between sub-groups. The low overall iodine status when bouillon was reported to have been consumed at least 6 times in the previous week by over 90% of respondent WRA [27], suggests that at the time of the survey, bouillon was not consistently being produced with iodised salt. This suggestion is supported by a study conducted in the two years preceding the survey which reported that only six out of thirteen tested bouillon brands were produced using salt with ≥15 mg/kg iodine [41].

The three surveys included in this study were designed with similar questionnaire modules allowing for the same regression model to be applied and for a comparison of findings between countries. Surveys were also planned with the input of national partners to reflect and provide an evidence base for the programme context in each country through appropriate stratification. The analyses could only be applied to variables collected during the surveys and could not account for any additional factors. A limitation to interpretation of some of the data is that the WRA respondent for the dietary diversity and frequency of food consumption questions was not always the respondent who provided the urine sample for iodine analysis, meaning that respondent data were used as a proxy for typical WRA practices in some cases. In addition, all analyses were based on casual spot urine samples which cannot be considered to reflect individual status. It was not feasible, or recommended, under survey conditions to collect the repeat urine collections or 24 h collections needed to reliably estimate individual iodine status [42].

## 5. Conclusions

This manuscript highlights the importance of the association between household salt iodine and iodine status among WRA and the fact that the national MUIC may hide large differences in iodine status according to location, residence type, vulnerability to poverty, and access to adequately iodised household salt. These disparities were evident to some extent in all three countries presented here, but were particularly marked in India and Ghana, although no sub-group of WRA in these two countries was found to be iodine deficient. Having data for different strata and other sub-groups facilitates decisions on the appropriateness of national salt iodine regulations, which in this case appear to be optimal in India but are recommended for review in Ghana, to introduce an acceptable range with an upper limit. In Senegal the fact that iodine status was relatively poor or else deficient among all population groups, leads to the recommendation to expand USI implementing guidelines to include enforcement of the use of adequately iodised salt by the processed food and condiment industry to improve iodine intake. This is also recommended in Ghana so that all main sources of dietary salt are iodised and salt iodine regulations can be established in accordance with this.

The influence of different factors on iodine status by sub-national area described in this paper emphasises the importance of collection and purposeful analysis of representative sub-national data by national programmes for achieving optimal iodine nutrition. Such data can be used to focus strategies as needed and ensure access to adequate iodine among all segments of the population. Designing surveys with standardised modules, programme-related stratification, and using multiple variable regression as part of the data analysis, can improve the utility of this sub-national evidence base. These data also provide a baseline from which to monitor the impact of any revisions in programme strategy.

## Figures and Tables

**Table 1:** Overview of the survey design, response rate, and respondent characteristics for each country.

Country	Year	Target Sample Size—HHs (HHs/PSU)	Sample Design			Response Rate (%)		Respondent Characteristics (%)	
			Target WRA for Urine Samples	Stratification	Sampling Scheme	Completed Interviews	Urinary Iodine Result ^1^	Female	WRA (% 15–17 yo)
India	2014–2015	6048 (12)	All WRA present in every second HH	12 strata: Urban/Rural by 6 zones: North, North-East, East, West, Central, South	Cross-sectional cluster, PPS within strata	94.5	86.5	91.3	82.2 (0.7)
Ghana	2015	2112 (16)	All WRA present in every HH	4 strata: North, Mid, South-non-producing, South-salt-producing	Cross-sectional cluster, PPS within strata	91.3	79.9	83.8	61.4 (0.0)
Senegal	2014	1968 (16)	One WRA where present in every HH	3 strata: Urban, Rural-non-salt producing, Rural-salt-producing	Cross-sectional cluster, PPS within strata	98.8	85.4	99.0	90.2 (1.4)

HH, household. PPS, probability proportional to size. PSU, primary sampling unit. WRA, woman of reproductive age (15–49 years old). yo, years old. ^1^ Response rate based on number of valid urinary iodine analysis results compared with the number of eligible WRA present in households targeted for urine collection at the time of the survey. The number of WRA not included for urine sample collection due to reporting being pregnant or possibly pregnant was 387 in India, 155 in Ghana, and 358 in Senegal.

**Table 2:** Regression analyses of factors associated with urinary iodine concentration (UIC) µg/L—India.

Variable	Level	Number Samples	Median	IQR	Single Variable Model—UIC µg/L			Multiple Variable Model—UIC µg/L		
					Mean	95% CI	*p*-Value	Relative to Reference	95% CI	*p*-Value (Variable Effect)
Strata	South—urban	201	147	72, 244	183	158, 208	<0.001			<0.001
	South—rural	202	162	78, 250	186	163, 209		1.1	0.9, 1.5	
	West—urban	188	177	96, 282	208	181, 235		1.2	0.9, 1.5	
	West—rural	204	147	81, 265	183	161, 206		1.1	0.9, 1.4	
	Central—urban	189	141	81, 226	181	155, 207		1.0	0.8, 1.2	
	Central—rural	190	115	605, 243	168	139, 197		0.9	0.7, 1.2	
	North—urban	157	224 ^a^	162, 309	246	223, 268		1.5 ^a^	1.2, 1.9	
	North—rural	176	169	103, 316	207	179, 236		1.3	1.0, 1.6	
	East—urban	204	162	90, 304	212	179, 244		1.1	0.9, 1.4	
	East—rural	192	155	67, 273	194	162, 225		1.1	0.8, 1.4	
	North East—urban	264	155	84, 246	192	164, 221		1.0	0.8, 1.3	
	North East—rural	253	136	69, 197	162	131, 193		0.9	0.7, 1.1	
Residence type	Rural	1217	149	73, 255	182	171, 194	0.002	Not included in multiple variable analysis		
	Urban	1203	168	92, 278	202	190, 213				
MPI education	High (deprived)	551	155	82, 238	179	166, 192	0.162			0.354
	Low	1869	160	82, 275	196	186, 205		1.1	1.0, 1.2	
MPI health	High (deprived)	672	160	79, 253	187	173, 201	0.223			0.683
	Low	1748	158	82, 268	194	184, 203		1.0	0.9, 1.1	
MPI living standards	High (deprived)	1449	149	73, 246	181	171, 191	<0.001			0.349
	Low	968	177	98, 289	208	196, 219		1.1	1.0, 1.2	
Heard of iodine deficiency	No	1046	149	70, 255	180	170, 191	<0.001			<0.001
	Yes	1374	165	89, 274	200	189, 211		1.2	1.1, 1.3	
Dietary diversity	Not diverse	1594	151	76, 256	185	176, 193	0.001			0.015
	Diverse	826	175	98, 281	206	191, 220		1.1	1.0, 1.2	
Household salt iodine content (mg/kg)	<5	193	112	45, 185	144	123, 166	<0.001			<0.001
	5–14.9	342	123 ^a^	61, 211	160	144, 176		1.3 ^a^	1.1, 1.6	
	≥15	1876	168 ^a^	91, 279	202	193, 211		1.6 ^a^	1.3, 2.0	
Age	<25	724	165	84, 283	201	186, 216	0.662			0.239
	25–29	457	152	81, 250	189	174, 204		0.9	0.8, 1.0	
	30–34	406	162	88, 248	188	173, 202		1.0	0.9, 1.1	
	35–49	833	155	77, 253	187	176, 198		0.9	0.8, 1.0	
BMI	<18.5	443	145	66, 251	180	165, 195	0.036			0.145
	18.5–25.0	1363	161 ^a^	85, 265	193	184, 203		1.1 ^a^	1.0, 1.2	
	>25.0	584	162 ^a^	89, 277	199	184, 213		1.1	1.0, 1.3	

CI, confidence interval. IQR, inter-quartile range. MPI, multi-dimensional poverty index. UIC, urinary iodine concentration. ^a^ Superscript letter indicates a significant difference to the reference value (first listed level) *p* < 0.05.

**Table 3:** Regression analyses of factors associated with urinary iodine concentration (UIC) µg/L—Ghana.

Variable	Level	Number Samples	Median	IQR	Single Variable Model—UIC µg/L			Multiple Variable Model—UIC µg/L		
					Mean	95% CI	*p*-Value	Relative to Reference	95% CI	*p*-Value (Variable Effect)
Strata	South-salt-producing	338	217	125, 352	265	228, 302	<0.001			<0.001
	North	378	168 ^a^	87, 291	228	185, 271		0.8	0.6, 1.0	
	Mid	298	174	115, 293	20	191, 248		0.8	0.7, 1.0	
	South-non-salt-producing	258	316 ^a^	187, 467	363	322, 403		1.2	1.0, 1.5	
Residence type	Rural	492	169	92, 291	224	188, 260	0.008			0.099
	Urban	780	221	138, 361	278	254, 302		1.2	1.0, 1.4	
MPI education	High (deprived)	572	181	93, 331	239	211, 268	0.002			0.376
	Low	700	217	136, 346	270	250, 290		1.1	0.9, 1.2	
MPI health	High (deprived)	765	185	107, 318	246	219, 272	0.008			0.222
	Low	502	216	138, 360	271	251, 291		1.1	1.0, 1.2	
MPI living standards	High (deprived)	1145	195	115, 333	253	232, 274	0.002			0.196
	Low	125	246	151, 386	293	261, 326		1.1	1.0, 1.3	
Heard of iodine deficiency	No	814	191	112, 318	250	225, 275	0.054			0.779
	Yes	458	216	134, 353	269	244, 293		1.0	0.9, 1.1	
Dietary diversity	Not diverse	473	195	126, 343	260	235, 285	0.848			0.995
	Diverse	799	204	119, 337	256	232, 281		1.0	0. 9, 1.1	
Household salt iodine content (mg/kg)	<5	423	180	110, 301	234	208, 260	<0.001			<0.001
	5–14.9	384	173	105, 312	226	196, 256		1.0	0.9, 1.1	
	≥15	301	291 ^a^	173, 447	334	304, 364		1.4 ^a^	1.2, 1.6	
Age	<25	443	202	130, 342	263	234, 292	0.705			0.073
	25–29	229	186	111, 329	250	222, 279		0.9 ^a^	0.8, 1.0	
	30–34	207	188	112, 339	254	220, 289		0.8 ^a^	0.7, 1.0	
	35–49	393	216	116, 343	258	231, 285		0.9 ^a^	0. 8, 1.0	
Tomato paste intake (g in past week)	0	238	163	84, 261	206	176, 235	<0.001			0.029
	<200	418	211 ^a^	130, 367	281	252, 310		1.1	1.0, 1.3	
	≥200	531	197 ^a^	124, 342	258	235, 282		1.2 ^a^	1.1, 1.4	
Bouillon intake (g in past week)	0	179	200	122, 328	244	215,273	0.003			0.023
	<20	691	216	131, 361	277	253, 300		1.2 ^a^	1.1, 1.3	
	≥20	366	168	97, 286	221	191, 251		1.0	0.8, 1.2	
Instant noodle intake	No	1055	194	120, 333	251	232, 271	0.064			0.436
	Yes	217	225	117, 400	289	249, 329		1.1	0.9, 1.2	

CI, confidence interval. IQR, inter-quartile range. MPI, multi-dimensional poverty index. UIC, urinary iodine concentration. ^a^ Superscript letter indicates a significant difference to the reference value (first listed level) *p* < 0.05.

**Table 4:** Regression analyses of factors associated with urinary iodine concentration (UIC) µg/L—Senegal.

Variable	Level	Number Samples	Median	IQR	Single Variable Model—UIC µg/L			Multiple Variable Model—UIC µg/L		
					Mean	95% CI	*p*-Value	Relative to Reference	95% CI	*p*-Value (Variable Effect)
Strata	Rural-salt-producing	462	109	48, 168	129	112, 146	0.174			0.210
	Rural-non-salt-producing	437	83	42, 147	115	95, 136		0.8	0.7, 1.1	
	Urban	409	112	50, 189	140	125, 155		1.1	0.8, 1.4	
Residence type	Rural	899	83	42, 147	115	95, 136	0.074	Not included in multiple variable analysis		
	Urban	409	112	50, 189	140	125, 155				
MPI education	High (deprived)	936	94	41, 162	124	110, 138	0.133			0.465
	Low	372	111	56, 183	138	122, 155		1	0.9, 1.4	
MPI health	High (deprived)	865	90	42, 164	125	110, 141	0.626			0.701
	Low	435	113	51, 176	135	119, 150		1.0	0.8, 1.2	
MPI living standards	High (deprived)	975	98	43, 164	127	111, 142	0.470			0.485
	Low	323	108	50, 178	133	114, 152		0.9	0.6, 1.2	
Heard of iodine deficiency	No	698	90	42, 162	125	109, 142	0.067			0.079
	Yes	610	113	52, 177	134	122, 147		1.2	1.0, 1.5	
Dietary diversity	Not diverse	440	97	43, 165	126	107, 146	0.770			0.329
	Diverse	868	103	46, 172	130	117, 143		0.9	0.8, 1.1	
Household salt iodine content (mg/kg)	<5	363	84	36, 141	107	90, 125	0.043			0.292
	5–14.9	502	85	44, 149	118	102, 134		1.1	0.8, 1.4	
	≥15	313	123 ^a^	56, 201	149	131, 168		1.3	0.9, 1.7	
Age	<25	471	107	44, 170	134	117, 151	0.542			0.360
	25–29	290	91	40, 170	128	108, 149		0.9	0.7, 1.2	
	30–34	205	95	44, 162	117	100, 134		0.8	0.6, 1.1	
	35–49	342	104	53, 171	129	112, 147		1.0	0.8, 1.3	
Bouillon intake (g in past week)	0	42	85	40, 114	91	70, 112	0.454			0.761
	<20	205	114	51, 167	124	106, 143		1.1	0.7, 1.8	
	≥20	1059	99	44, 171	131	117, 145		1.2	0.8, 1.7	

CI, confidence interval. IQR, inter-quartile range. MPI, multi-dimensional poverty index. UIC, urinary iodine concentration. ^a^ Superscript letter indicates a significant difference to the reference value (first listed level) *p* < 0.05.

## Disclaimer

J.K. is an independent consultant. The opinions and statements in this article are those of the author and may not reflect official IGN policies. R.K. is a UNICEF staff member. The opinions and statements in this article are those of the author and may not reflect official UNICEF policies. J.G. is the Executive Director of the Iodine Global Network. The opinions and statements in this article are those of the author and may not reflect official IGN policies. G.S.G is a GAIN staff member. The opinions and statements in this article are those of the author and may not reflect official GAIN policies.
